# Philosophical Bioethics in the Policy Arena: A Response to Open Peer Commentaries on “Just Policy? An Ethical Analysis of Early Intervention Policy Guidance”

**DOI:** 10.1080/15265161.2018.1549291

**Published:** 2019-01-24

**Authors:** Rose Mortimer, Alex McKeown, Ilina Singh

**Affiliations:** University of Oxford

We thank the authors of the open peer commentaries (OPCs) for their thoughtful and illuminating contribution to the debate. There were lots of topics that we wanted to explore in our target article, but that had to be cut from earlier drafts since there was insufficient space to include them, or because we worried that reference to other relevant literatures might detract from the main argument of the piece. Consequently, we are very grateful to the OPC authors for introducing some of these, including Care Ethics (Ho and colleagues 2018); the role of neuroscience and epigenetics in driving early intervention (EI) policy forward (Pentecost and Meloni 2018); and the influence of background conditions of social injustice in contributing to the extent to which parents may be less able to exercise responsibility and choice (Brighouse and Swift 2018). Elsewhere, the OPC authors have introduced new literatures or ideas that we had not considered, such as Latham’s intriguing discussion of experimental philosophy in the role of blame and praise, and we are excited by this opportunity to take the debate in new directions.

Considering the six OPCs as a set, we feel that the criticisms or claims made can be divided into two groups. First, there are those that critique or at times misunderstand the role we pose for bioethics in the public policy arena, or the methods that bioethicists should use in this space (Latham 2018; Brighouse and Swift 2018; Ho et al. 2018). Second, there are those who contribute to our discussion of responsibility attribution in the context of EI, proposing different ways of considering responsibility, additional factors that are relevant to attributing responsibility, or discussing other actors who perhaps should take on more responsibility (Canavera et al. 2018; Pentecost and Meloni 2018; Wallis and Weiss 2018). We consider each of these sets of OPCs in turn, though some respondents touch on both topics and as such there will be an element of overlap within our discussion.

First, however, we briefly reiterate the goal and purpose of our article, since it seems that some of the commentators may have misunderstood what we were seeking to achieve by conducting an ethical analysis of this kind.

## THE GOAL AND PURPOSE OF OUR ARTICLE

In our target article, we sought to achieve two aims. First, we used a corpus of policy documents in order to explore the ethical arguments inherent in the contemporary EI agenda in the United Kingdom. Second, we used the preceding discussion as an illustrative case study in order to motivate a debate about the role of philosophical bioethics in policy guidance and politics more generally.

In the ethical analysis, we sought to analyze the arguments that we found within the corpus of policy documents, so as to understand the values and assumptions that underlie the EI agenda. Therefore, while we may appear to have overlooked alternative approaches to the ethical issue in question—for example, the “systematic injustice” approach introduced by Brighouse and Swift—this is not necessarily because we were unaware of these alternative perspectives but because they were not represented within the policy corpus. We do not deny that these alternative perspectives have value. However, the fact that we did not discuss them does not undermine the legitimacy of our approach, since it was never our stated aim to provide the kind of complete and detailed ethical analysis that Brighouse and Swift recommend. Our article offered a snapshot of the EI policy landscape focusing on a corpus of key documents, rather than a thorough review of all EI policy documents and all possible responses to them.

## THE ROLE OF BIOETHICS IN PUBLIC POLICY

In the second half of the target article, we discussed the role that bioethicists can play in assisting policymakers, particularly in the context of EI, but also more generally. We made a number of claims about the value that bioethicists can bring to this enterprise, including that they can:

- Draw attention to relevant debates in the bioethics literature.
- Examine the values underlying policy proposals and explore where ethical principles conflict.
- Reveal where policy is overly rhetorical and lacks clarity.
- Bring to light the practical and theoretical implications of holding certain views.
- Consider what it would mean to put these policies into practice, particularly whether the “real world” outcome is likely to be “ethical.”
- Consider whether the values inherent in policy proposals adequately represent, or at least consider, the plurality of values existing in society.

We sought to be clear about what bioethics can do, but also about what it can’t or shouldn’t do. We argued that philosophical bioethics need not work as a logic monitoring service for politicians, and we maintained that the test of a good policy is much more complex than simply assessing the quality of the argument that led to a specific conclusion; arguments are important, but far more important is the way that a particular policy plays out in the “real world.”

## PHILOSOPHICAL RIGOR IDENTIFIES THE “TRUTH”?

Importantly, we do not suggest that a little more philosophical rigor in policy would reveal the one Truth to which everyone would have to then assent. To the contrary, we agree with Latham that the truth can be “tyrannical” if it leads one to become intolerant of disagreement and to disregard alternative perspectives. In conducting this analysis of policy guidance we definitely do not wish to subvert the plurality of views held across society. The value of scrutinizing these seemingly unarguable policy claims is precisely to reveal how views other than those endorsed by the government are acceptable. In doing so, we hope to return some agency to citizens whose values and goals do not perfectly align with those of the state.

Rather than finding and arguing for one “truth,” we envisage our role—and the role of philosophers working in policy more generally—as closer to what John Locke, in *An Essay Concerning Human Understanding,* calls the “under-labourer”: “clearing the ground a little, and removing some of the rubbish that lies in the way to knowledge.” (Locke 1836, 4–5) In doing this work, we identify the most salient points in policy, sweeping away emotive rhetoric and seeking to critically examine the core claims being made and the values and assumptions underlying them. This does not mean that our parsing of the language will point the way toward the one right or true answer.

## PHILOSOPHICAL ANALYSIS HELPS POLICY TO BEST REPRESENT PUBLIC OPINION?

Brighouse and Swift also slightly mischaracterize the role we claim for bioethics in public policy. They believe our claim is that philosophical analysis may help policy to better represent public opinion. In response, they argue that philosophical analysis may often revise and contradict the views of those in society, and therefore “close interrogation of underlying values and assumptions may just as well lead one away from public opinion as toward it, and it may do nothing to resolve disagreements.”

We did not intend to suggest that interrogating the values underlying policies will necessarily resolve disagreements, nor that policy should represent public opinion in the sense that it will advocate the views of the majority public. Rather, we argue that the policymaking process should be sufficiently nuanced to take into account the varied views of citizens, and to try as far as possible to respect these and fairly weigh them up when deciding upon the best solution. As we have made clear in the preceding discussion, when policy is overly rhetorical or emotive it may at times appear that there really is only one ethical value at play, or one solution that any reasonable person would agree with. As such, it is easy for policymakers to overlook the fact that reasonable people actually value a large range of different things, and that the particular vision of the good childhood or the good family reflected in policy is just one account of a contested and complex idea. To imply that it is the only account is to overlook or deny the valid perspectives of various individuals in society. Of course, it may be impossible to resolve disagreements between publics, and it may be wrong to advocate the views of some citizens when these views or values are ethically problematic. However, the plurality and richness of public opinion should be recognized and respected; good philosophical analysis can help policy to achieve this.

## BIOETHICS IS JUST FOR PHILOSOPHERS?

The role of bioethics within policymaking is not limited to the kind of philosophical analysis conducted within our article. Indeed, in order to further explore “what matters” to various different groups in society, bioethicists with different skills and backgrounds may be better placed to collect and analyze data from the real world, thus enhancing our argument through empirical findings. The field of “empirical bioethics” is growing steadily, and often borrows methodology from social science, anthropology, or law, involving tools such as interviews or participant observation, combining descriptive work with philosophical argument (see Ives and colleagues 2017). As Pentecost and Meloni rightly claim within their OPC, “the ‘bios’ of bioethics is rapidly changing” and new methods may need to be developed in order to understand the social life of the science that drives policy and practice. Given this, we absolutely agree with Ho and colleagues that bioethical work is not limited to the kind of philosophical bioethics done here, and that social scientists, as well as other kinds of scientists, can of course be bioethicists.

Ho and colleagues are also right to suggest that we did not give much space to discussing our method of analyzing the data, simply describing it as “normative analysis.” However, we hope it became clear throughout the article that the kind of work we undertook was an ethical analysis of EI policy documents, whereby we explored the arguments made within policy documents and the ethical values underlying them, and carefully assessed these arguments within the context of relevant philosophical theories. Ho and colleagues claim that it is important to distinguish between foundational and nonfoundational normative inquiry, and they rightly state that the work we have conducted is nonfoundational, since we sought to explicate and evaluate the content of substantive moral norms already accepted as applicable in the given context—moral norms such as justice and responsibility. We called this “philosophical bioethics” because of the focus on argument, and because much of the literature we drew upon originated within philosophy. We chose this method of inquiry in order to view the ethical issue in question through a new lens, and hopefully to bring new ideas or insights to the debate. But in doing so, we do not seek to undermine or discount the valuable work of those in the social science community—some of whom identify as bioethicists—who have long studied the ethical implications of EI and the nature of the relationship between families and the state.

## RESPONSIBILITY IN EARLY INTERVENTION

Canavera and colleagues, Pentecost and Meloni, and Wallis and Weiss all consider the role of responsibility, blame, and guilt in the context of EI. They propose different ways of considering responsibility, highlight additional factors that are relevant to attributing responsibility, and discuss other actors who perhaps should take on more responsibility for child well-being and addressing injustice.

Canavera and colleagues make the point that responsibility for EI services advocacy should be shared between multidisciplinary stakeholders, particularly including health care professionals such as pediatricians**.** We entirely agree with this claim. In our methodology section we explain that “the final corpus was chosen to include a diverse sample of publishers … EI is a cross-sector initiative in the United Kingdom”; this is demonstrated by the fact that the documents we analyzed originated from a number of different government departments, all of which are interested in EI from their own angle or perspective. However, while responsibility for advocating for EI services certainly does cross disciplines, the way in which EI services and policy attribute responsibility—and hence blame—for child outcomes almost always places it quite firmly with parents. This was a key finding of our analysis.

In our article we focus on “the vast majority of EI programs [that] work with parents or pregnant women.” These are the kinds of EI programs typically discussed within the policy documents that we analyzed, and they focus mostly on child well-being in terms of mental health and character, in the very early years of life—typically between conception and when the child first starts school. But as Canavera and colleagues rightly point out, there are many other kinds of EI that can take place in the context of a child’s life, and some of these concern older children, or children with serious physical health problems—in these cases, it may be more often the responsibility of doctors and other medical professionals to care for the child. While parenting interventions are recommended for some mental health conditions like conduct disorder or attention-deficit hyperactivity disorder (ADHD), it is of course impossible for “good parenting” to have any major impact on the cause or outcome of an illness like leukemia.

Indeed, the distinction between EI for physical health and that for mental health is pertinent in the context of the OPC written by Wallis and Weis. These authors make the point that it is important to distinguish primary and secondary forms of EI. Primary prevention aims to prevent the development of a condition in “at risk” populations, whereas secondary prevention aims to identify a condition early to mitigate the development of negative sequelae. They argue that “by failing to distinguish between these EI program types and the prevention each offers, Mortimer, McKeown, and Singh have muddied the discussion of parental, clinician, and state responsibilities in EI.”

We agree that the distinction between primary and secondary intervention matters, and it could have been more clearly discussed in our article. However, we wonder whether the distinction is significant in quite the way that the authors of this OPC imply, given the problematics of diagnosis in the context of childhood behavioral disorders in particular. In medicine, the boundary between primary and secondary intervention is generally said to depend upon the absence or presence of clear symptoms of disorder. This boundary is often demarcated by diagnosis, such that interventions post diagnosis are called “secondary” interventions. But diagnostic uncertainty characterizes much of psychiatry (Singh and Wessely 2015). Lacking good biomarkers, psychiatric diagnosis is most often based on behavioral assessment and/or observations (Singh and Rose 2009). A condition like ADHD, for example, exists on a continuum with “normal” behavior; therefore, it can be difficult to distinguish “normal” and “abnormal” hyperactive and disruptive behaviors (Parens and Johnston 2009). The controversy over the use of medications in children with such behaviors points to the problematics of ADHD diagnosis, and the blurred lines between “primary” and “secondary” intervention in such cases.

Indeed, in the case of ADHD, once a child is diagnosed, responsibility isn’t simply handed over to medical professionals; parents must make decisions about medication on weekends, and manage the child’s behavior not only through medication but also through the home environment and parenting style. Conditions like ADHD are of particular interest to politicians and others who advocate for early childhood intervention because of the extent to which the behaviors exhibited by the child are viewed as in some way “deviant,” and the concern that they prevent an individual from gaining the skills and character traits necessary for future good citizenship. This, policy claims, perpetuates inequality, because some children are born with conditions and traits that make it more difficult for them to achieve a good life.

Here it is perhaps helpful to briefly comment upon the role of parents in promoting social justice, and the relationship we discuss between parents and the state. In our article we suggested that while policy situates “good parenting” as the best route to promoting equality and social justice, this was not necessarily the case, because of the ways in which aspirational parenting may give some children an unfair advantage over their peers. However, in saying this, we do not seek to set up any kind of inherent opposition between the family and justice; indeed, we agree with Brighouse and Swift that the family is in most cases a part of social justice. Rather, we simply seek to show how, by focusing only on neglectful or abusive parenting, policy documents fail to recognize some of the ways in which the aspirational parenting that they advocate may also advance inequality. In doing so, we question whether it is really brute inequality that these policy documents seek to address, or whether there are additional assumptions about the meaning of good parenting that can be unpicked. Our own view is that in many cases, being a “good enough” parent is sufficient, and it is potentially demoralizing or unfair to place too many expectations upon parents required to raise the next generation of ideal citizens. Of course we can imagine ideal circumstances for childrearing and the ideal products of these circumstances, but it is easy to forget that the “real world” is far removed from these philosophical thought experiments, meaning that it may at times be pragmatic to settle for “good enough.”

Finally, Pentecost and Meloni rightly suggest that while we make reference to how findings from neuroscience and epigenetics have come to shape the policy debate, we do not make this a focus of our analysis in the article. We were not able to give sufficient space to discussing the ways in which findings from neuroscience and epigenetics have shaped the EI policy debate, and particularly how the move from genetics to epigenetics has recast the nature/nurture debate and forced us to consider new accounts of responsibility. We were able to touch on this only briefly in our methodology section, and we are very grateful to these authors for shining more light on this important aspect of the discussion around responsibility and early childhood intervention. ▪
